# Effect of Different Catholytes on the Removal of Sulfate/Sulfide and Electricity Generation in Sulfide-Oxidizing Fuel Cell

**DOI:** 10.3390/molecules28176309

**Published:** 2023-08-29

**Authors:** Thi Quynh Hoa Kieu, Thi Yen Nguyen, Chi Linh Do

**Affiliations:** 1 Institute of Biotechnology, Vietnam Academy of Science and Technology, 18 Hoang Quoc Viet, Cau Giay, Hanoi 100000, Vietnam; 2 Faculty of Biotechnology, Graduate University of Science and Technology, Vietnam Academy of Science and Technology, 18 Hoang Quoc Viet, Cau Giay, Hanoi 100000, Vietnam; 3 Institute of Material Sciences, Vietnam Academy of Science and Technology, 18 Hoang Quoc Viet, Cau Giay, Hanoi 100000, Vietnam

**Keywords:** catholyte, ferricyanide, phosphate buffer, dissolved oxygen, sulfide-oxidizing fuel cell, sulfate-reducing bacteria

## Abstract

Microbial fuel cells are one of the alternative methods that generate green, renewable sources of energy from wastewater. In this study, a new bio-electrochemical system called the sulfide-oxidizing fuel cell (SOFC) is developed for the simultaneous removal of sulfide/sulfide and electricity generation. To improve the application capacity of the SOFC, a system combining sulfate-reducing and sulfide-oxidizing processes for sulfate/sulfide removal and electricity generation was designed. Key factors influencing the sulfide-removal efficiency and electricity-generation capacity of the SOFC are the anolytes and catholytes. The sulfide produced from the sulfate-reducing process is thought to play the key role of an electron mediator (anolyte), which transfers electrons to the electrode to produce electricity. Sulfide can be removed in the anodic chamber of the SOFC when it is oxidized to the element sulfur (S°) through the biochemical reaction at the anode. The performance of wastewater treatment for sulfate/sulfide removal and electricity generation was evaluated by using different catholytes (dissolved oxygen in deionized water, a phosphate buffer, and ferricyanide). The results showed that the sulfate-removal efficiency is 92 ± 1.2% during a 95-day operation. A high sulfide-removal efficiency of 93.5 ± 1.2 and 83.7 ± 2% and power density of 18.5 ± 1.1 and 15.2 ± 1.2 mW/m^2^ were obtained with ferricyanide and phosphate buffers as the catholyte, respectively, which is about 2.6 and 2.1 times higher than dissolved oxygen being used as a catholyte, respectively. These results indicated that cathode electron acceptors have a direct effect on the performance of the treatment system. The sulfide-removal efficiency and power density of the phosphate buffer SOFC were only slightly less than the ferricyanide SOFC. Therefore, a phosphate buffer could serve as a low-cost and effective pH buffer for practical applications, especially for wastewater treatment. The results presented in this study clearly revealed that the integrated treatment system can be effectively applied for sulfate/sulfide removal and electricity generation simultaneously.

## 1. Introduction

Wastewater containing sulfate/sulfide is a typical kind of corrosive, odorous pollutant and is toxic to human health and living organisms [1,2,3]. Under anaerobic conditions, sulfate-reducing bacteria (SRB) utilize organic compounds as carbon and energy and sulfate as the terminal electron acceptor for sulfide production (H_2_S, HS^−^, and S^2−^), according to Equation (1) [4,5]. Therefore, sulfide is also ubiquitously present in these types of wastewater:

2 CH_2_O + SO_4_^2−^ → H_2_S + 2 HCO_3_^−^ (1)

The treatment processes for sulfate/sulfide are still limited; especially, sulfide is a hazardous substance and needs to be removed from wastewater before being discharged into the environment [6,7]. Conventional wastewater treatment methods such as oxidization by chloride (Cl^−^), potassium permanganate (KMnO_4_), hydrogen peroxide (H_2_O_2_) [8,9], air injection, and oxidation by sulfide-oxidizing bacteria (SOB) can be used to prevent the formation of sulfide [10]. Although these methods are able to effectively remove sulfide, they share a common limitation of high energy and chemical consumption, which would result in high operating costs and large amounts of sludge. Therefore, it is necessary to seek novel methods to simultaneously remove sulfate/sulfide from wastewater to consume less energy and chemicals and to produce less sludge [11].

In order to solve these problems, a microbial fuel cell (MFC), a novel method that has been considered a promising method in producing electricity and reducing operating costs and toxic byproducts compared with conventional treatments, is proposed in this study. Recently, several studies have investigated the uses of MFCs for the treatment of sulfide and simultaneous electricity generation [2,12,13,14].

MFCs are bioelectrochemical systems that can generate electricity via microbial metabolism while removing pollutants such as organic compounds, sulfate, sulfide, nitrate, etc. [15]. In MFCs, microbes switch their metabolic pathway to use electrodes as electron acceptors, oxidize pollutants (electron donors) in the anode, and produce protons and electrons. These electrons travel through an external circuit to the cathode, producing electricity. The protons generated in the anode also diffuse to the cathode across the proton-exchange membrane (PEM). For each electron that is produced, an equivalent proton must be transported to the cathode through the electrolyte, which sustains the current flow. Finally, the electrons and protons combine with oxygen (an electron acceptor) in the cathode to produce water [16,17,18].

Because electricity generation can stimulate the microbial oxidation of the pollutants, MFCs are potentially useful in bioremediation. Therefore, improving the power density is one of the greatest challenges for the practical applications of MFCs [15]. 

Many efforts have been made to improve power generation, including (i) the microbial oxidation of the substrate; (ii) electron transfer from the microbe to the anode; (iii) electron transfer through the circuit, including the external circuit; (iv) proton diffusion from the anode chamber to the cathode chamber; and (v) oxygen supply and reduction at the cathode [15,19,20]. Among these factors, the efficiency of (ii) electron transfer from the microbe to the anode and (v) oxygen supply and reduction at the cathode have been noted to be the most critical factors controlling the power density and contaminant-removal efficiency of an MFC. It was discovered that the current density and power output could be greatly enhanced by the addition of electron mediators into the anode. Mediators play an important role in electron transport for those microbes that are unable to transfer the electrons to the anode. However, synthetic exogenous mediators are toxic to microorganisms and expensive. The toxicity and instability of synthetic exogenous mediators (dyes and metalorganics) limit their applications in MFCs [21]. Therefore, the application of MFCs with endogenous mediators produced by microorganisms such as sulfide, iron, pyocyanin, etc., is advantageous in wastewater treatment and power generation [22]. In the present study, the sulfide produced in the sulfate process by SRB acts not only as an endogenous electron mediator but also as an electron donor to oxidize sulfide into nontoxic sulfur that is recoverable by precipitation [23].

One major problem associated with dual-chambered MFCs is low redox potential in the cathodic chamber [24]. Protons are of great significance for cathode reaction in MFCs. This proton limitation in the power production of an MFC that lacks buffer capacity in the catholyte, such as dissolved oxygen, has been demonstrated [25,26]. Hence, to improve the redox potential on the cathode, it may be advantageous to use a catholyte with higher cathodic redox potential, such as phosphate buffer, dichromate, ferricyanide, permanganate, etc. [27]. However, there are no direct comparisons made in the same system with different catholytes. Therefore, the use of sulfide as an anolyte and ferricyanide, phosphate buffer, and dissolved oxygen as catholytes in the same MFC has never been performed prior to this study.

The objectives of this study were to (1) design an integrated SRBR–SOFC system to treat sulfate/sulfide contaminated wastewater and electricity generation; (2) evaluate the efficiency of sulfate removal in the SRBR; and (3) investigate the effect of different catholytes (ferricyanide, phosphate buffer, and deionized water (dissolved oxygen)) on sulfide removal and electricity generation in the same SOFC.

## 2. Results

### 2.1. Overall Performance of SRBR–SOFC System

#### Efficiency of Sulfate Removal in the SRBR

In order to illustrate the capability of the SRBR–SOFC system in sulfate, sulfide removal, and electricity generation, the integrated SRBR–SOFC system was operated continuously with the COD/SO_4_^2−^ ratio of 2 and sulfate concentration of 1300 mg/L. The experiment was run for 95 days with three different catholytes (deionized water, phosphate buffer, and ferricyanide). The synthetic sulfate-contaminated wastewater (see Section 4.1.2) was continuously fed at the bottom of the SRBR by a peristaltic pump with an HRT of 72 h. The effluent of the SRBR, containing a sulfide concentration of 3.16 ± 6.9 mg/L, was continuously supplied using a peristaltic pump into the anodic chamber of the SOFC at an HRT of 24 h. 

**Figure 1:**
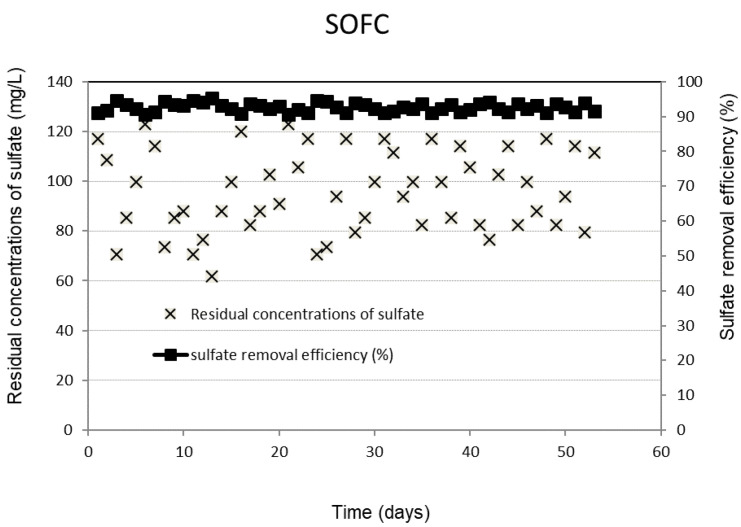
Removal efficiency and residual concentrations of sulfate in SRBR with time.

Figure 1 shows the daily performance outcomes of the SRBR, including the removal efficiencies and residual concentrations of sulfate under the selected conditions. The obtained results showed that the concentrations of sulfate decreased significantly after the first seven days of the experiment. The lag period observed in the initial phase was due to the slow initiation of activities of SRB. Once the consortium of sulfate-reducing bacteria in the treatment system is fully established, the system is capable of biodegrading the organic matter and reducing sulfate with a relatively high removal rate and less sensitivity to the influent concentration fluctuations. The sulfate removal efficiency was stable during the 95-day operation. Sulfate was converted by 92 ± 1.2% of the initial concentration to sulfide of 316 ± 6.9 mg/L. The final sulfate concentration existing in the SRBR effluent is 98 ± 15.6 mg/L (Figure 1). 

### 2.2. Performance Comparison of SOFC Using Different Catholytes

#### 2.2.1. Effect of Three Different Catholytes on Sulfide Removal in SOFC

**Figure 2:**
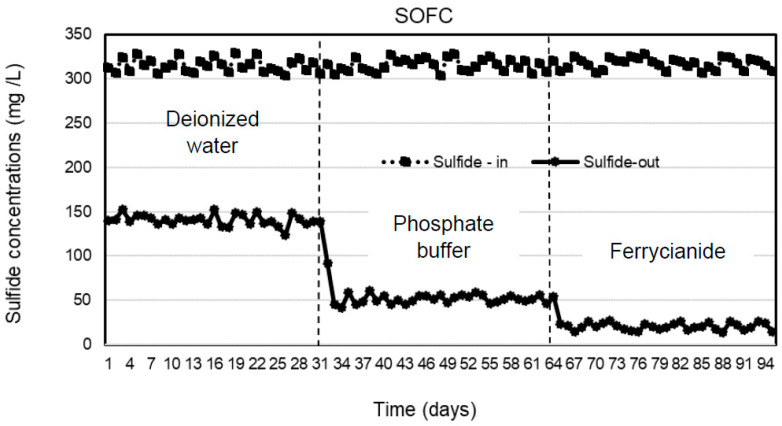
Sulfide concentrations in the anodic influent and effluent of SOFC with deionized water, phosphate buffer, and ferricyanide as catholytes with time.

**Figure 3:**
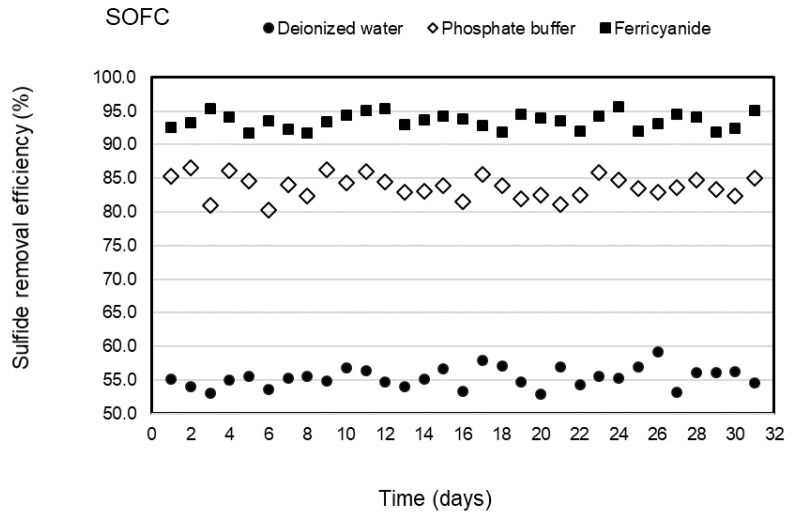
Sulfide removal efficiencies of SOFC with deionized water, phosphate buffer, and ferricyanide as catholytes with time.

Three types of catholytes, deionized water, phosphate buffer, and ferricyanide, were investigated for sulfide removal and electricity generation in the cathodic chamber of the SOFC system under continuous operation. The anode chamber was filled with the effluent of the SRBR at an HRT of 24 h during the 31-day operation of each catholyte with the average sulfide concentrations of 316 ± 6.9 mg/L. Concentrations of sulfide in the anodic influent and effluent of the SOFC are shown in Figure 2. The results indicated that sulfide was removed stably in the anode chamber within the 31-day operation for each catholyte. Sulfide-removal efficiencies of 55.4 ± 1.5, 83.7 ± 2, and 93.5 ± 1.2% were observed with deionized water, phosphate buffer, and ferricyanide as catholytes, respectively. Higher average sulfide-removal efficiencies of 83.7 ± 2 and 93.5 ± 1.2% were observed, respectively, with phosphate buffer and ferricyanide as catholytes as compared to deionized water (55.4 ± 1.5%) (Figure 3). The average sulfide concentrations remaining in the anode effluent were 141 ± 6.3 mg/L for deionized water, 51 ± 4.9 mg/L for phosphate buffer, and 20.5 ± 3.8 mg/L for ferricyanide (Figure 2). The results showed that the sulfide removal in the anodic chamber was affected by the different catholytes. This indicated that more availability of electron acceptors in the cathodic chamber enhanced the rate of oxidation of sulfide in the anodic chamber of the SOFC. 

#### 2.2.2. The Effects of Three Different Catholytes on Electricity Generation in SOFC

In this treatment system, cathode electron acceptors had direct effects not only on sulfide removal but also on electricity generation. This means that the sulfide present in the anode chamber was oxidized, releasing electrons and protons to the anode during the SOFC operation, resulting in electricity production. 

**Figure 4:**
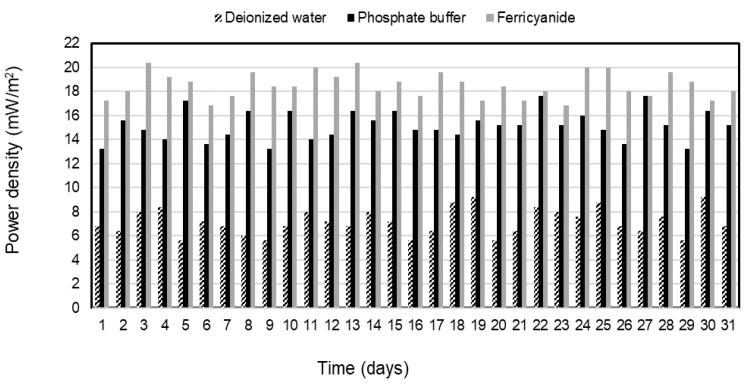
Power density with deionized water, phosphate buffer, and ferricyanide as catholytes with time.

Electricity was generated continuously from the sulfide oxidation process of the SOFC during the 31-day operation of each catholyte. The average power densities of 7.2 ± 1.1, 15.2 ± 1.2, and 18.5 ± 1.1 mW/m^2^ were observed and remained stable for deionized water, phosphate buffer, and ferricyanide as catholytes in the SOFC, respectively (Figure 4). The electricity was constantly generated in the deionized water SOFC after 7 days of operation due to the slow initiation of SRB activities. Over the same period, sulfate in SRRB was converted by about 91% of the initial concentration to sulfide of 312 mg/L in the treatment system, whereas the high-power density was generated immediately following the phosphate buffer and ferricyanide SOFC without any adaptation phase. This suggested that the performance of the SOFC was mainly governed by the catholyte used and made faster consumption of electrons on the cathode and better oxidization of sulfide by the anode electrode with this catholyte. The power density with ferricyanide and phosphate buffer as electron acceptors demonstrated superior performance when compared with water and other chemical catholytes, as reported in previous studies [28,29]. The results (Figure 2, Figure 3 and Figure 4) indicated that higher sulfide-removal efficiency in the SOFC leads to higher electricity generation.

**Figure 5:**
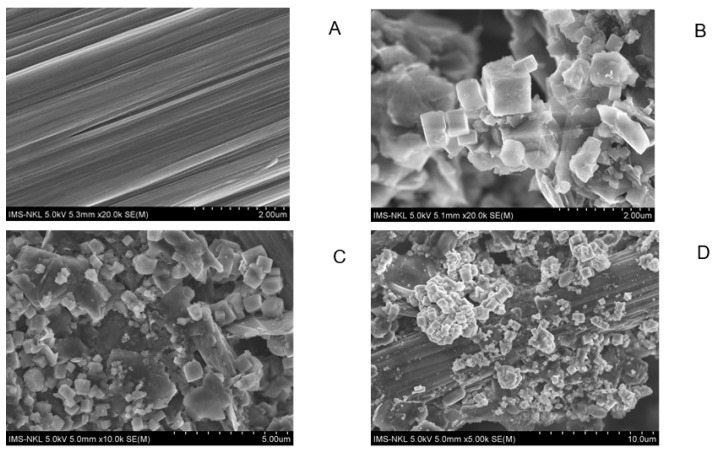
SEM image of anode before (**A**) and after SOFC operation with different catholytes, (**B**) deionized water, (**C**) phosphate buffer, and (**D**) ferricyanide.

**Figure 6:**
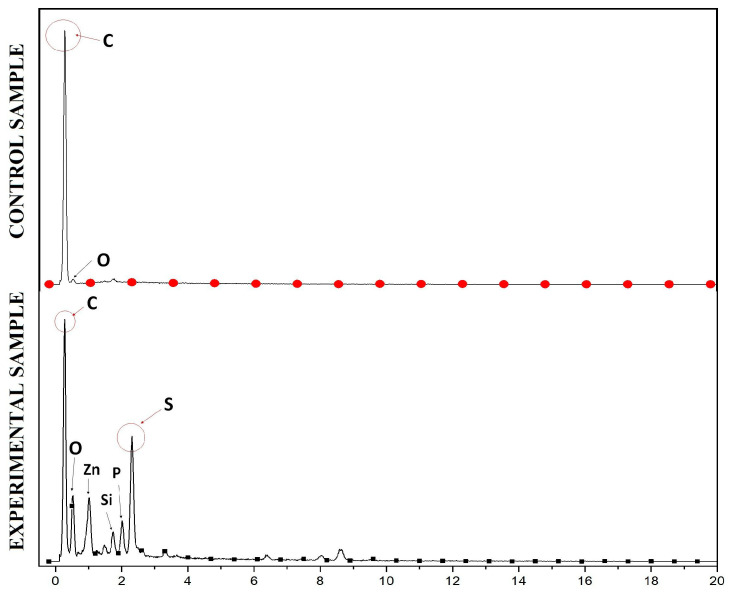
EDS analysis of the anode surface before (**above**) and after (**below**) SOFC operation with ferricyanide catholytes.

The results of anode surface analysis by SEM at the end of the operation of the deionized water, phosphate buffer, and ferricyanide SOFCs are shown in Figure 5. The EDS analysis of the anode surface before and after SOFC operation with ferricyanide catholytes is presented in Figure 6. The amount of sulfur attached to the anode significantly increased during the operation of the SOFCs. The results of SEM-EDS also revealed the attachment of solid plaques, which were most likely elemental sulfur. The accumulation of sulfur on the anode may have decreased the conductivity of the anode, thereby increasing the ohmic loss of the SOFC system. Moreover, they might hinder the effect of mass transport of fresh sulfide ions to the anode electrode if SOFCs are operated for a long period of time. This result was similar to previous studies [30]. To prevent this problem, the sulfur accumulation should be recovered for the sustainable operation of SOFCs.

## 3. Discussion

The sulfide produced biologically based on the organic carbon-oxidizing and sulfate-reducing processes by sulfate-reducing bacteria in the SRBR plays the key role of electron mediator in the SOFC, which transfers electrons to the electrode to produce electricity, and an additional amount of synthetic exogenous mediators, which are toxic and expensive compounds, is not necessary. Therefore, using sulfide produced from the sulfate-reducing process in the SRBR as an electron donor has been considered a promising method to reduce operating costs and toxic byproducts compared with traditional treatments. Sulfide can be removed in the anodic chamber of the SOFC when it is oxidized to element sulfur (S°) through the bio-electrochemical reaction at the anode. In this process, sulfide is an electron donor, and the anode acts as the final electron acceptor. The addition of an extraneous electron acceptor, such as nitrate, is not necessary.

The presence of sulfate in the anode media resulted in negative effects on sulfide oxidation and electricity generation in MFCs. Ref. [31] reported that low concentrations of sulfate (≤1470 mg/L) benefited MFC efficiency, while higher sulfate presence blocked the sulfide oxidization and electricity generation. Therefore, to remove pollutants from wastewater effectively and generate stable electricity in long-term operation, a sulfate-reducing bioreactor (SRBR) should be coupled with a sulfide-oxidizing fuel cell (SOFC). 

Oxygen is a catholyte in an MFC as its use is cost-effective, has no waste product, and has unlimited availability [32]. As mentioned above, protons are of great significance for cathode reactions in an MFC. This proton limitation in the power production of an MFC that lacks buffer capacity in the catholyte as dissolved oxygen in deionized water has been reported. The performance of these MFCs was limited by poor oxygen-reducing catalytic activity at the cathode [25]. This problem could be solved by either the addition of protons (acidified water solution) or buffered solution as catholytes to maintain pH at a near-neutral range in MFCs [33]. 

In order to eliminate proton limitations and to increase power generation, various researchers have tested the performance of MFCs using different catholytes with high cathodic redox potentials, such as phosphate buffer, potassium ferricyanide, dichromate, permanganate, etc. [34,35]. The addition of catholytes led to faster reduction kinetics by overcoming the activation energy barrier, thus minimizing cathodic activation loss. The cathode activation overpotential can be reduced by employing a catholyte with a higher redox potential. The results showed that these catholytes (potassium ferricyanide, dichromate, and permanganate) were relatively higher in power density compared to an MFC with dissolved oxygen [36]. However, each of the cathode electron acceptors had its own advantages and disadvantages. Therefore, it is important to evaluate the effectiveness of different catholytes in the same SOFC and understand their advantages and disadvantages.

In the present study, the sulfate removal efficiency of the SRBR was 92 ± 1.2%. The obtained results showed that at an initial COD/SO_4_^2−^ ratio of 2 with a sulfate concentration of 1300 mg/L, most of the sulfate in the influent was converted to sulfide. A similar result was demonstrated by Ref. [37] with sulfate removal efficiency over 91% at COD/SO_4_^2−^ ≥ 2.5 for sulfate concentrations of 1960 mg/L. Sulfide removal and power production from the effluent of the SRBR containing a sulfide concentration of 316 ± 6.9 mg/L were investigated in the SOFC with different catholytes (dissolved oxygen, phosphate buffer, and ferricyanide). The average sulfide removal of 55.4 ± 1.5, 83.7 ± 2, and 93.5 ± 1.2% was observed with dissolved oxygen, phosphate buffer, and ferricyanide as catholytes, respectively. The sulfide removal was affected by catholytes due to the difference in redox potential. Higher sulfide removal was observed in the SOFCs with ferricyanide and phosphate buffer as catholytes, which implied that more availability of electron acceptors in the cathodic chamber enhanced the rate of oxidation of sulfide in the anodic chamber. The lowest performance of sulfide removal was observed with dissolved oxygen as a catholyte due to slower reduction kinetics at the cathode. The SOFC generated maximum sustainable power densities of 7.2 ± 1.1, 15.2 ± 1.2, and 18.5 ± 1.1 mW/m^2^ with dissolved oxygen, phosphate buffer, and ferricyanide as catholytes, respectively. Using different catholytes, the power density increased by 2.6 times (ferricyanide) and 2.1 times (phosphate buffer) as compared to using dissolved oxygen. The results showed that catholytes have a significant effect on sulfide removal and electricity generation in the SOFC. Ref. [35] reported that voltage decreased from 0.525 V to 0.32 V over a period of 330–422 h in the cathodic chamber, which was supplied with a non-buffered solution. At the same time, the pH value increased from 7.15 to 8.82, which revealed a strong dependence of proton levels on electricity generation. This proton limitation has been shown by Ref. [34]. The author reported that the maximum volumetric power generated in the MFC with ferricyanide, dichromate, permanganate, and persulfate were 2.3, 2.2, 3.4, and 7.1 times higher than that obtained with dissolved oxygen (water) as the catholyte, respectively. 

In comparison, ferricyanide is used as an electron acceptor to produce higher power than systems using dissolved oxygen catholytes. Ferricyanide solution is used in cathodic mediums in SOFCs to substitute oxygen as a catholyte due to low overpotential. The cathode activation overpotential can be reduced by employing catholytes with higher redox potential [38,39]. However, the use of ferricyanide is expensive and environmentally harmful, which restricts their application for the commercialization of MFC technology. Therefore, other low-cost and nontoxic catholytes, such as phosphate-buffered solutions, have also been used to complete the cathodic reduction reactions in MFCs [40,41]. The good performance of the SOFC using phosphate as the pH buffer and proton carrier indicated that a phosphate buffer could serve as a low-cost and effective pH buffer for practical applications, especially for wastewater treatment. A phosphate buffer solution has been commonly used in MFC studies [42,43] to maintain a suitable pH for electricity-generating bacteria and/or to increase the solution conductivity. In the absence of a buffer, the pH in the cathode chambers was changed, resulting in a much lower power density [44]. 

In this study, an SOFC with phosphate buffer (50 mM PBS) catholyte produced only slightly less sulfide removal and power density than that obtained using ferricyanide in a two-chamber SOFC and 2.1 times higher than that from a dissolved oxygen SOFC (Figure 3 and Figure 4). 

## 4. Materials and Methods

**Figure 7:**
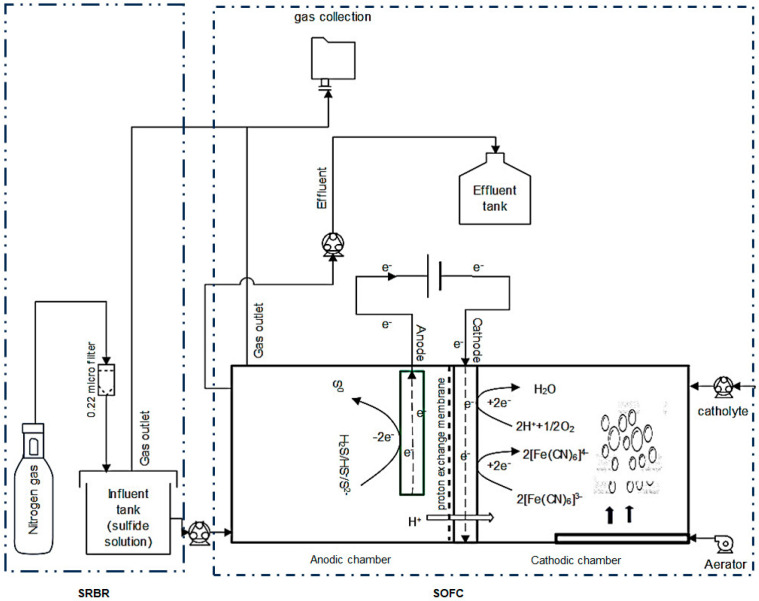
Configuration of a sulfate-reducing bioreactor (SRBR) integrated with a sulfide-oxidizing fuel cell (SOFC).

In this study, a two-chamber SOFC system similar to MFC technology, coupled with a sulfate-reducing bioreactor (SRBR), was designed, as shown in Figure 7. The SRBR–SOFC consists of two identical components: (1) a sulfate-reducing bioreactor (SRBR) to reduce sulfate to sulfide (sulfide solution) and (2) a sulfide-oxidizing fuel cell (SOFC) to subsequently oxidize sulfide to element sulfur (S°). The integrated use of the SRBR and SOFC can remove sulfate and sulfide simultaneously with electricity generation. 

### 4.1. Sulfate-Reducing Bioreactor (SRBR)

#### 4.1.1. The Inoculum

A consortium of sulfate-reducing bacteria (SRB) was enriched from anaerobic sludge rich in sulfide from a crude oil tanker in Vung Tau, Vietnam, and used as the inoculum. This culture was cultivated under anaerobic conditions [45] using Postgate’s medium B [46] with a slight modification (KH_2_PO_4_ 0.5; NH_4_Cl 1.0; Na_2_SO_4_ 1.0; MgSO_4_·7H_2_O 2.0; Sodium lactate 3.2; Yeast extract 0.5; FeSO_4_·7H_2_O 0.01; ascorbic acid 0.1; thioglycolic acid 0.1; pH = 7.0 ± 0.2). To achieve a high density of SRB (1 × 10^8^ cells/mL), 10% of the volume of the culture was replaced by fresh medium every three weeks. 

#### 4.1.2. SRBR and Operating Conditions

Sulfate reduction was carried out in continuous anaerobic sulfate-reducing bioreactor (SRBR) in an up-flow mode. This reactor was fabricated from glass, having a working volume of 250 mL. 

The SRBR was started up by directly inoculating 10% (*v*/*v*) of the enriched SRB consortium containing 1 × 10^8^ cells/mL into the SRBR fed with synthetic wastewater containing sulfate and sodium lactate as an electron acceptor and electron donor, respectively. The composition of the synthetic wastewater (g/L) consisted of KH_2_PO_4_, 0.5; NH_4_Cl; Na_2_SO_4_, 1.9; MgSO_4_·7H_2_O, 0.06; and sodium lactate, 4.28 (in COD); pH = 7.0 ± 0.2. After inoculation with the enriched SRB consortium, the reactor was purged by N_2_ gas to provide the anaerobic condition. The synthetic wastewater was continuously injected into the bottom of the SRBR by a peristaltic pump (Ismatec SA, Zuerich, Switzerland) with a hydraulic retention time (HRT) of 72 h for 95 days at ambient room temperature (25 ± 2 °C) after reaching a dynamic equilibrium (7-day lag period) (≥90% reduction in sulfate was achieved). 

### 4.2. Sulfide-Oxidizing Fuel Cell (SOFC)

#### 4.2.1. Design and Fabrication of SOFC

A two-chambered SOFC (sulfide-oxidizing fuel cell) system was designed. The SOFC consists of two identical chambers (working volume of 80 mL each): (i) an anode chamber, where electrochemical oxidation of sulfide on an anode surface derives electrons and protons, and (ii) a cathode chamber, where catholytes (dissolved oxygen in deionized water, phosphate buffer, and ferricyanide) react with the released protons. This SOFC was made with mica acrylic plates. These chambers were divided by a proton-exchange membrane (PEM). For the cathode, a catalyst layer was prepared by brushing the Pt/C 40 wt.% catalyst ink on a carbon cloth with an active area of around 10 cm^2^. The cathodic electrode was fabricated by hot pressing the catalyst layer on a Nafion 117 membrane with the following conditions: temperature of 135 °C, duration time of 180 s, and pressure of 21 kg/cm^2^. For the anode, a plain carbon cloth (1071 HBC—USA) was utilized with a working area of about 5 cm^2^.

#### 4.2.2. SOFC Operation Conditions

To investigate the feasibility of sulfide removal and the electricity generation capacity of the SOFC, the effluent of SRBR containing a sulfide concentration of 316 ± 6.9 mg/L was fed continuously into the anode chamber of SOFC by a peristaltic pump (Ismatec SA, Zuerich, Switzerland) with an HRT of 24 h. 

The SOFC system was operated under continuous mode with three different catholyte conditions, where deionized water, phosphate buffer, and ferricyanide were successively used as catholytes. In each catholyte condition, the SOFC was operated with a frequency of 31 days. In the first operating condition, deionized water catholyte was fed into the cathode chamber of the SOFC. Then, the continuous operation of the SOFC was carried out for the next 31 days with 50 mM phosphate buffer (NaH_2_PO_4_, 2.45 g/L; Na_2_HPO_4_ 4.58 g/L and 0.31 g/L NH_4_Cl; 0.13 g/L KCl, pH 6.9). For the last operating condition, the cathode chamber was switched from phosphate buffer to potassium ferricyanide solution (50 mM K_3_Fe (CN)_6_, pH 6.9) with a frequency of 31 days. 

Before and after each experiment, all the electrodes were first immersed in 1 M NaOH and 1 M HCl, respectively, for 24 h, and then in sterile deionized water for 48 h to remove microbial residues on the electrodes’ surfaces. Dissolved oxygen (DO) was maintained at about 6.8 ± 0.4 mg/L by continuously sparging air through a diffuser connected to an aerator (an aquarium pump) at a rate of 3 mL/min. To maintain the pH and chemical composition for sufficient deoxidation in the cathode chamber, the cathodic solution was recirculated to improve the agitation at flow rate of 150 mL/h and freshly changed with a cathodic electron solution every week. All catholyte solutions were prepared aseptically to avoid contamination.

#### 4.2.3. Experimental Design of Integrated SRBR–SOFC System

The purpose of the experiment design for this study was to identify the effect of different catholytes on the performance of an SOFC in a sulfide-contaminated wastewater treatment system. The system was operated for 95 days with three catholytes (deionized water, phosphate buffer, and ferricyanide). Each catholyte was operated for 31 days. The SOFC system was operated continuously with HRT of 72 h for SRBR and 24 h for SOFC at COD/SO_4_^2−^ ratio of 2 and sulfate concentration of 1300 mg/L without the addition of external substrates or electron transport mediator during the experiment. After reaching a dynamic equilibrium (7 days), the sulfate and sulfide concentrations of both influent and effluent and the current and voltage were measured continuously. These results were used for estimating the sulfate, sulfide-removal efficiencies, and power density of the system, as detailed in the following section. The influent and effluents from SRBR and SOFC were sampled to investigate removal efficiencies of sulfate and sulfide every day. Power density was monitored every day to check for changes in the electricity generation efficiency during the operation.

#### 4.2.4. Experimental Analysis

Influent and effluent samples of SRBR and SOFC were collected over time for sulfate and sulfide measurement. Before each analysis, the samples were filtered through a 0.45 µm nitrocellulose membrane syringe filter. Sulfate was measured using the turbidimetric method based on the addition of barium chloride to form a colloidal suspension of barium sulfate at 420 nm [47]. Sulfide (H_2_S, HS^−^, and S^2−^) was measured at 480 nm according to Cord–Ruwish method based on CuS precipitation [48]. Power density (P, mW/m^2^) was counted according to P = IU/A, where I (A) is current, U (mV) is voltage, and A (m^2^) is the surface area of the cathode. Surfaces of the clean anode and the used anode were analyzed using scanning electron microscopy (SEM) (HITACHI S-4800). For SEM examination, samples were first immersed in glutaraldehyde (2.5%, 60 min) and then washed with phosphate buffer (0.1 M, pH 7.0, 3 times). Finally, the samples were treated with critical point drying to dehydrate the biological tissues and coated with Pt.

## 5. Conclusions

The key factor identified to affect the sulfide removal and electricity generation of the wastewater system is the catholyte. Different types of catholytes led to different sulfide-removal efficiencies and electricity generation capabilities for the SRBR–SOFC system. Effective sulfide removal and electricity generation can be achieved in a two-chamber SOFC using phosphate buffer and ferricyanide as catholytes rather than using dissolved oxygen in dissolved oxygen as the catholyte. Among all the electron acceptors, ferricyanide demonstrated the highest sulfide-removal efficiency and power generation. However, the use of ferricyanide is expensive and toxic to the environment, and its application on a large scale is not sustainable. In this study, the SOFC with a phosphate buffer (50 mM PBS) catholyte produced only slightly less sulfide-removal efficiency and power density than those obtained using ferricyanide in a two-chamber SOFC, and higher than those of a dissolved oxygen SOFC. The good performance of the SOFC using phosphate as a pH buffer and proton carrier indicated that a phosphate buffer could serve as a low-cost and effective pH buffer for practical applications, especially for wastewater treatment. There remains further interest in increasing effective power generation by optimizing the phosphate buffer concentration at the cathode.

## Data Availability

All data generated or analyzed during this study are included in this article.
